# A Clival Mass Posing a Diagnostic Challenge: Pituitary Adenoma With Rathke Cleft Cyst Mimicking Chordoma

**DOI:** 10.7759/cureus.102137

**Published:** 2026-01-23

**Authors:** Eliany Leon Figueredo, Lauren Lopez, Ruben E Diaz Samada, Idalberto Luis Fernandez Eng, Lilian A Maturell Rojas, Pablo Estorino

**Affiliations:** 1 Internal Medicine, Englewood Health Physician Network, Englewood, USA; 2 Neurology, Neurology Consultants of Central Florida, Kissimmee, USA; 3 Internal Medicine, Hospital Provincial Clínico Quirúrgico Docente "Saturnino Lora Torres", Santiago de Cuba, CUB; 4 Emergency Medicine, Hospital Universitario de la Ribera, Valencia, ESP; 5 Internal Medicine, Universidad de Ciencias Médicas de Guantánamo, Guantánamo, CUB; 6 Medicine, Educational Commission for Foreign Medical Graduates (ECFMG), La Porte, USA

**Keywords:** chordoma, clival lesion, collision tumors, histopathology, pituitary adenoma, rathke's cleft cyst, skull base tumor

## Abstract

Ectopic or extrasellar pituitary adenomas (PAs) in the clival region are extremely rare and can mimic aggressive skull base tumors on imaging, posing a diagnostic challenge. We present the case of a 54-year-old woman with a six-month history of severe, pulsatile headaches, without visual, endocrine, or neurological deficits. Laboratory evaluation revealed mild metabolic abnormalities. Imaging demonstrated a large, destructive clival mass with heterogeneous enhancement, bone erosion, lateral extension into the cavernous sinus and Meckel’s cave, and encasement of the left internal carotid artery, strongly suggestive of a clival chordoma. The patient underwent staged surgical resection, including endoscopic endonasal and left orbitozygomatic approaches. Intraoperative frozen section suggested pituitary tissue, and final histopathology confirmed a PA associated with a Rathke cleft cyst. This case highlights the diagnostic challenges of clival lesions: radiologic aggressiveness may not reflect the underlying pathology, and clinical symptoms may be minimal despite extensive tumor burden. Definitive diagnosis requires integration of imaging, surgical findings, and histopathology. Multidisciplinary collaboration is essential to guide appropriate management, avoid misclassification, and optimize outcomes.

## Introduction

Pituitary tumors (PTs) comprise a heterogeneous group of lesions arising in the sellar and parasellar regions, including adenohypophyseal, neurohypophyseal, and non-pituitary pathologies. Pituitary adenomas (PAs) represent the most common subtype, originating from adenohypophyseal cells, and account for approximately 15% of all primary brain tumors and nearly 25% of benign primary brain tumors. In the current World Health Organization classification, PAs are also referred to as pituitary neuroendocrine tumors (PitNETs), reflecting their neuroendocrine differentiation; however, the term “PA” remains widely used in clinical practice. The sellar (or intrasellar) region corresponds to the sella turcica, the bony cavity that houses the pituitary gland. PTs may extend laterally beyond the sella and posteriorly infiltrate the clivus [1]. Depending on tumor size, growth pattern, and invasiveness, PTs may extend into adjacent anatomical compartments, giving rise to distinct topographic classifications with important diagnostic and surgical implications [2].

Ectopic PitNETs, an extremely rare entity, are defined as adenohypophyseal tumors located entirely outside the sella turcica, without anatomical or radiological continuity with the intrasellar pituitary gland [3]. These tumors may involve the suprasellar, parasellar, or clival regions. In some rare cases, two distinct neoplasms can coexist in close proximity within the sellar or clival region, referred to as collision tumors. These tumors may consist of a PA adjacent to another lesion, such as a meningioma, craniopharyngioma, or Rathke cleft cyst, and may complicate both radiologic interpretation and surgical planning. Collision tumors underscore the need for careful histopathological evaluation to accurately identify each component and guide appropriate management [4].

Common symptoms of ectopic PTs in the sphenoid sinus and clivus include nasal congestion, headache, impaired vision, cerebrospinal fluid leakage, cranial nerve palsy, and signs of endocrine dysfunction, such as fatigue and decreased libido. These tumors can be larger and more invasive than lesions located in the cavernous sinus or suprasellar peri-infundibular region, leading to distinct clinical presentations [3].

The suprasellar region refers to the area superior to the diaphragma sellae and includes critical structures such as the optic chiasm, hypothalamus, pituitary stalk, and floor of the third ventricle. Parasellar extension denotes lateral growth into the cavernous sinuses, which contain the internal carotid artery and cranial nerves III, IV, V1, V2, and VI. Less commonly, PTs may extend posteriorly or inferiorly toward the clivus, sphenoid sinus, or prepontine cistern [4]. There are no distinguishing characteristics on MRI that could alert the physician to the presence of these ectopic tumors preoperatively. Clival PTs usually appear as heterogeneous, solid, expansile-enhancing masses, often associated with bone remodeling or erosion, and may occasionally demonstrate calcification [5].

Ectopic clival PTs can exhibit aggressive behavior, including bone invasion, which typically appears as areas of destruction on imaging. Tumor seeding and malignant transformation have also been reported, although the underlying mechanisms of such aggressiveness remain unclear. In some cases, invading muscles and encasing vessels can also be considered within the aggressiveness criteria [6]. Lesions arising in or extending into the sellar, suprasellar, and parasellar regions encompass a broad differential diagnosis, including other central skull base lesions such as chordoma, meningioma, chondrosarcoma, plasmacytoma, lymphoma, and metastasis [7].

Because these entities often share overlapping radiologic features and may present with aggressive-appearing bone destruction or cavernous sinus involvement, preoperative distinction based solely on imaging can be challenging. Given their atypical clinical presentation and nonspecific radiological findings, definitive diagnosis relies on postoperative histopathological and immunohistochemical analysis. Here, we present the case of a 54-year-old woman with a clival mass whose radiologic appearance was highly suggestive of a chordoma. Despite its aggressive imaging features, the final diagnosis was a PA associated with a Rathke cleft cyst, highlighting the limitations of imaging-based diagnosis and the critical role of histopathological confirmation.

## Case presentation

A 54-year-old woman with no significant past medical history presented for a routine annual physical examination. She reported a six-month history of severe, pulsatile headaches occurring more than three times per week, localized to the posterior head. The headaches often worsened at night and frequently awakened her from sleep. The pain reached a severity of 10/10. She denied nausea, vomiting, visual loss, or focal neurological deficits.

A complete laboratory evaluation, including serum hormone levels (thyroid-stimulating hormone, prolactin, luteinizing hormone, follicle-stimulating hormone, growth hormone, and adrenocorticotropic hormone) and metabolic parameters, was performed. All results were within normal limits except for the abnormalities summarized in Table 1, which included mild metabolic disturbances: prediabetic glucose levels, borderline high cholesterol, low vitamin D, and slightly elevated mean platelet volume.

**Table 1:** Abnormal laboratory findings at annual physical examination

Test	Result	Normal range	Interpretation
Mean platelet volume	12.6 fL	7.5-11.5 fL	Slightly high
Fasting glucose	111 mg/dL	70-99 mg/dL	Prediabetic
Hemoglobin A1c	5.70%	4.0-5.6%	Prediabetic
Total cholesterol	201 mg/dL	<200 mg/dL	Borderline high
Low-density lipoprotein	124 mg/dL	<100 mg/dL	High
Vitamin D	17.8 ng/mL	30-100 ng/mL	Low
Urinalysis - blood	Trace	Negative	Trace

**Figure 1:**
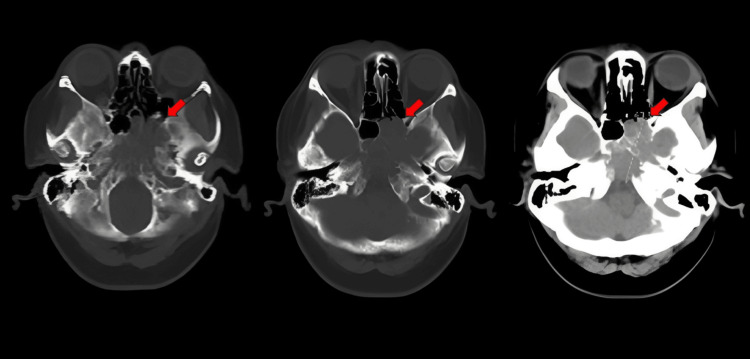
Preoperative non-contrast CT of the head showing a destructive clival mass (red arrows)

A non-contrast CT scan of the head revealed a large, destructive mass centered at the clivus, measuring 4.4 × 3.8 × 2.6 cm, with extension into the sellar/suprasellar region, adjacent left temporal lobe, sphenoid sinus, and prepontine cistern. No hemorrhage, mass effect, or hydrocephalus was present. The findings were concerning for an aggressive skull base neoplasm, and an MRI of the brain and internal auditory canals with contrast was recommended for further characterization (Figure 1).

**Figure 2:**
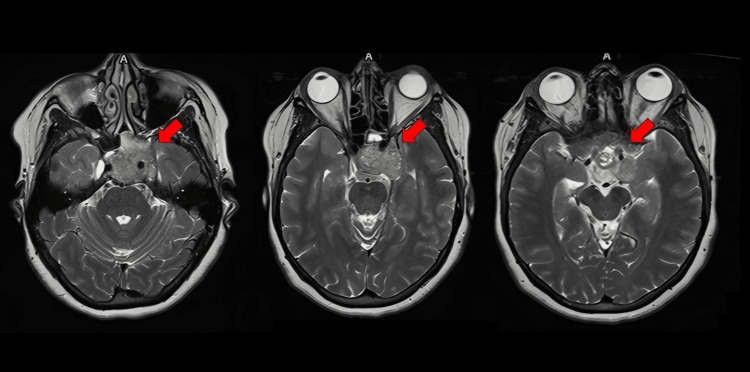
Preoperative axial MRI of the brain showing a large clival mass (red arrows)

MRI demonstrated a large, avidly enhancing clival mass measuring 4.0 × 3.5 × 3.3 cm, with posterior extension into the prepontine cistern abutting the basilar artery, lateral extension into the left cavernous sinus and Meckel’s cave with encasement of the left internal carotid artery, and superior extension into the suprasellar cistern adjacent to the left medial temporal lobe. There was no restricted diffusion, hemorrhage, or ventricular abnormality (Figure 2).

Neurosurgical evaluation suggested a clival chordoma, and the patient was referred to a skull base surgical team. As part of the multidisciplinary assessment, bilateral rigid nasal endoscopy was performed. Findings included a rightward deviated septum with a left bony spur, mild bilateral inferior turbinate hypertrophy, and mild mucosal edema, with no evidence of polyps. The patient was deemed a suitable candidate for endoscopic transsphenoidal resection of the clival mass, with the option for an expanded approach to address tumor extension into the cavernous sinus and Meckel’s cave using image-guided navigation. This decision was based on the lesion’s predominantly midline clival location, inferior extension, and the absence of extensive lateral cortical involvement, allowing safe access through an endonasal corridor. A transcranial approach was considered as a staged alternative in case of residual tumor or limited endonasal accessibility.

The patient underwent endoscopic transsphenoidal resection of the pituitary/skull base neoplasm, along with a craniotomy via an orbitozygomatic approach, secondary dural repair using graft material, and correction of the deviated nasal septum. The procedure was performed as part of a multidisciplinary skull base surgical plan to achieve maximal safe resection while preserving cranial base integrity and function.

Postoperative MRI of the pituitary gland with and without contrast revealed that the majority of the previously described mass had been resected, with residual tumor noted at the sella, left of the sella, and left suprasellar cistern, measuring up to 3.1 cm in maximum diameter. The residual tumor contacted both internal carotid arteries at the level of the cavernous sinuses. There was a slight rightward deviation of the infundibulum, with no mass effect on the optic chiasm. No acute ischemia or abnormal parenchymal enhancement was identified.

CT angiography of the head demonstrated postoperative debulking of the central portion of the clival mass, with a mild-to-moderate rim of residual tumor more prominent on the left and extension into the left cavernous sinus. Destructive clival changes persisted. All major intracranial arteries and venous structures appeared normal, with no evidence of aneurysm, arteriovenous malformation, vasospasm, or vascular injury.

**Figure 3:**
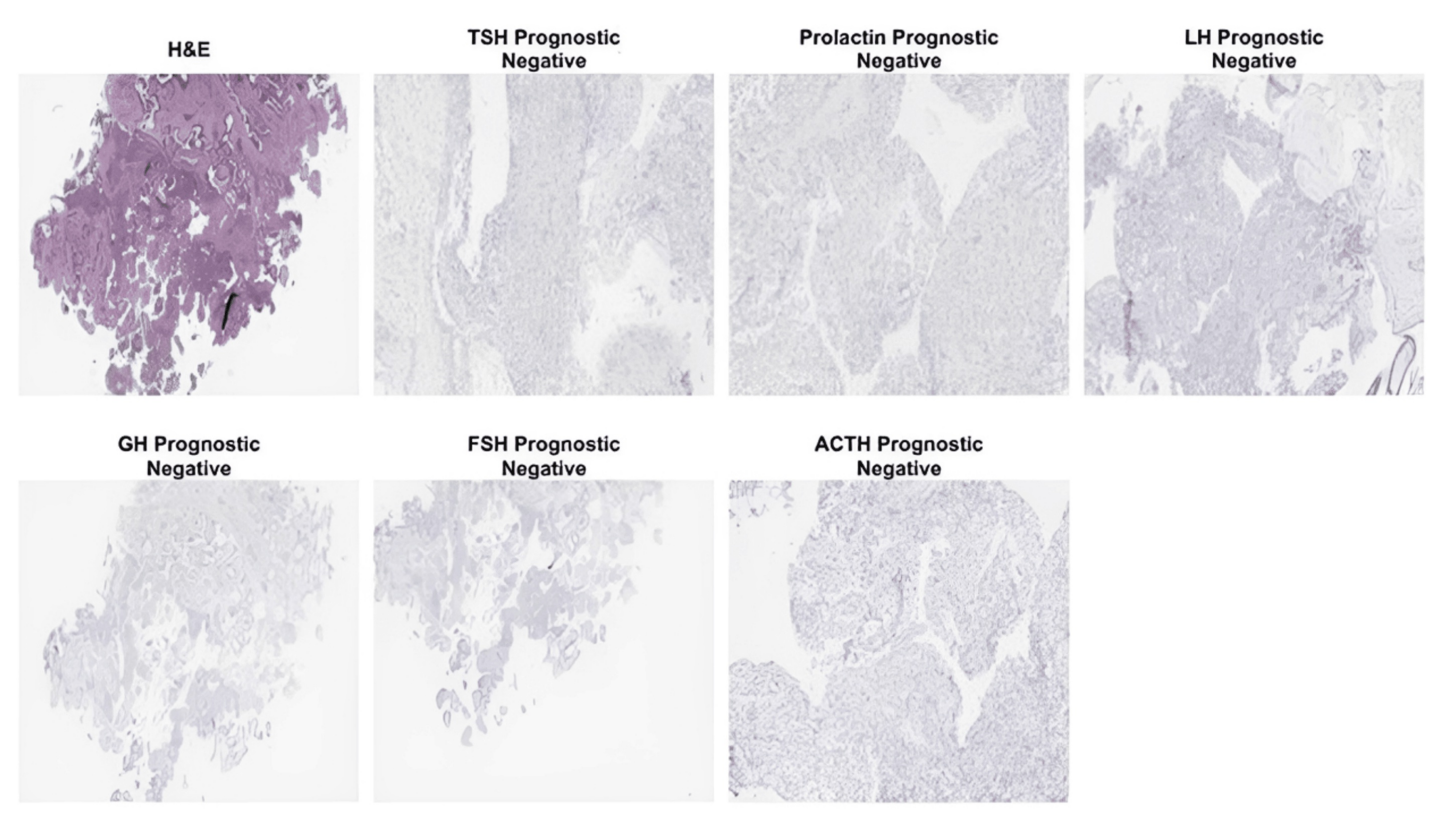
Histopathological and immunohistochemical findings H&E staining demonstrates a monomorphic adenohypophyseal neoplasm. Immunohistochemical staining for pituitary hormones, including TSH, prolactin, LH, FSH, GH, and ACTH, is negative, consistent with a non-functioning PA. These findings support the diagnosis and exclude hormone-secreting PTs. ACTH, adrenocorticotropic hormone; FSH, follicle-stimulating hormone; GH, growth hormone; LH, luteinizing hormone; PA, pituitary adenoma; PT, pituitary tumor; TSH, thyroid-stimulating hormone

Histopathological analysis clarified the true nature of the lesion. Intraoperative frozen section initially revealed only pituitary cells, suggesting a pituitary-origin process. Final evaluation of the excised specimen demonstrated a PA accompanied by a Rathke cleft cyst, indicating that the mass consisted of mixed sellar pathology rather than a primary clival neoplasm. Immunohistochemistry supported these findings: AE1/AE3 highlighted the epithelial lining of the Rathke cleft cyst, synaptophysin confirmed the presence of adenomatous pituitary tissue, S100 staining was negative, and the reticulin pattern further supported the diagnosis of PA. These combined results explained the intraoperative impressions and provided a definitive diagnosis that differed from the initial radiologic suspicion of chordoma (Figure 3).

**Figure 4:**
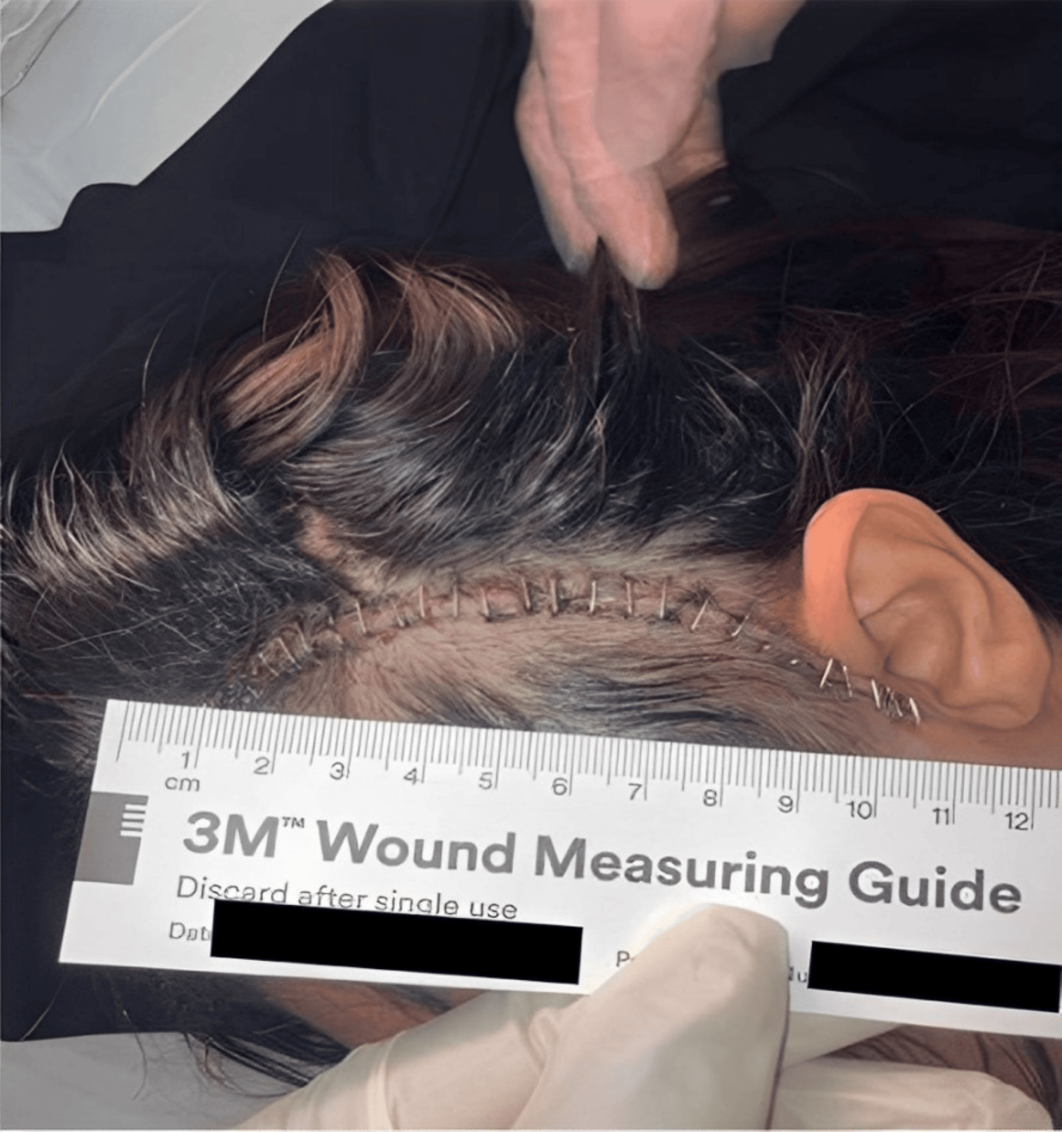
Postoperative photograph showing the left orbitozygomatic surgical incision following second-stage tumor resection

Given the extent of the residual mass, the patient subsequently underwent a second-stage resection via a left orbitozygomatic craniotomy to achieve maximal safe tumor debulking while preserving critical neurovascular structures (Figure 4).

Postoperatively, the patient underwent regular clinical and neurological evaluations. She reported significant improvement in headache frequency and intensity, with no recurrence of severe pulsatile headaches. Neurological examinations revealed no new focal deficits or cranial nerve dysfunction. Endocrinological assessment after surgery showed no evidence of new hypopituitarism, and pituitary hormone levels remained within normal limits. The patient continued outpatient follow-up with neurosurgery and endocrinology.

Postoperative MRI demonstrated expected postsurgical changes, including heterogeneous enhancement in the clival bed, a 1.8-cm rim-enhancing fluid collection, mild orbital and facial soft tissue swelling, persistent pneumocephalus, and a small left temporal subdural collection, with preservation of the pituitary gland, stalk, optic chiasm, and major intracranial vessels. At six months, follow-up MRI demonstrated stable postoperative findings, including a mildly deviated but normally enhancing pituitary stalk, a stable lobulated fluid collection near the left orbit, and unchanged nonspecific white matter FLAIR hyperintensities, with otherwise normal intracranial and orbital structures.

## Discussion

Pituitary region tumors are predominantly PAs, which exhibit sex-related patterns: women tend to present earlier with more microadenomas, while men more often develop larger, nonfunctioning lesions. Craniopharyngiomas account for less than 4% of cases, with the adamantinomatous subtype predominating and minor ethnic variations. Rare sellar tumors, such as meningiomas, chordomas, and posterior PTs, represent approximately 1% and often show male predominance [8]. PTs are a heterogeneous group of central nervous system lesions, usually benign, with an estimated prevalence of 16.7%. Their clinical presentation is variable: some cause prominent symptoms, while others produce subtle, slowly developing complaints that delay diagnosis. The most common symptom is headache (37-70%), typically related to dural stretching but not always correlated with tumor size. Large tumors can cause obstructive hydrocephalus, increasing intracranial pressure and resulting in headache, nausea, papilledema, visual disturbances, cognitive changes, poor coordination, or personality alterations. Specific tumor extensions may cause seizures, dementia, nasal obstruction, epistaxis, craniocervical instability, or brainstem compression [2,9]. Clival or extrasellar PAs are rare. Published cases often present with visual impairment, cranial nerve deficits, endocrine dysfunction, or nasal obstruction, whereas isolated headache is uncommon [10]. Our patient’s presentation with severe, pulsatile headaches as the sole symptom is therefore atypical, illustrating that significant radiologic tumor burden may occur without overt neurologic or endocrine signs.

The pathophysiology of ectopic PAs is not fully understood, but several mechanisms have been proposed. Ectopic PAs located along the embryologic migrational pathway of pituitary development are thought to arise from residual adenohypophyseal cells left behind during the ascent of Rathke’s pouch from the primitive oral ectoderm to the sella turcica. These remnants may persist along the nasopharynx, sphenoid sinus, suprasellar region, or clivus and later undergo neoplastic transformation. In contrast, ectopic PAs found outside this migrational tract are believed to result from alternative mechanisms, including aberrant differentiation of pluripotent cells, misplaced embryonic tissue, or secondary extension or displacement of sellar adenomas with subsequent loss of an apparent anatomical connection to the pituitary gland. These theories help explain the heterogeneity of ectopic PA locations and underscore why such lesions may present diagnostic challenges, particularly when arising in atypical skull base regions such as the clivus [3,5].

Collision tumors of the sellar region are rare entities defined by the coexistence of two histologically distinct lesions in close anatomical proximity without histological intermixture. The most frequently reported combination involves PAs and Rathke cleft cysts, although other associations have been described. The pathogenesis of these lesions remains unclear, with proposed mechanisms including coincidental development, shared embryologic origin, or local microenvironmental factors promoting the growth of adjacent but distinct lesions [4]. Clinically and radiologically, collision tumors pose a diagnostic challenge, as imaging findings often reflect the dominant component and may fail to reveal the presence of a second lesion preoperatively. In the present case, the absence of a clearly identifiable cystic component on MRI and the aggressive-appearing clival involvement obscured the coexistence of a Rathke cleft cyst, which was only recognized on histopathological examination.

The lesion’s radiologic appearance strongly favored a clival chordoma: a midline destructive lesion, heterogeneous enhancement, bone erosion, cavernous sinus encasement, and extension into adjacent compartments. These features are typical of chordomas and other skull base malignancies, including chondrosarcomas, making imaging-based distinction challenging. Cases of pituitary macroadenomas mimicking invasive skull base tumors have been reported, demonstrating that MRI can overestimate aggressiveness. This underscores a critical lesson: imaging alone cannot reliably differentiate chordomas from ectopic PAs, and misclassification could lead to inappropriate surgical planning, overtreatment, or altered follow-up strategies [11].

This aspect represents the core of the case: the radiologic appearance of the lesion was so highly suggestive of a clival chordoma that all initial evaluations, multidisciplinary discussions, and surgical planning were oriented toward managing a malignant skull base tumor. Clival chordoma carries a completely different prognosis, recurrence rate, treatment algorithm, and morbidity profile compared with PA. Therefore, the fact that a benign PA with an associated Rathke cleft cyst displayed such intense bone destruction, midline clival involvement, and cavernous sinus extension is clinically significant. It underscores the potential for profound diagnostic error when relying on imaging alone and highlights the indispensable role of histopathology in guiding correct management. In this case, tissue diagnosis did not merely clarify the pathology; it entirely changed the expected natural history, postoperative management, and long-term follow-up strategy.

PAs are generally benign, but a subset can behave aggressively by invading surrounding structures, particularly the cavernous sinus and internal carotid artery, which complicates surgical management and increases the likelihood of recurrence. Preoperative MRI using the Knosp classification is the most common tool to predict invasiveness, although it is known to produce false positives. Moreover, studies in other complex surgical contexts have shown that unplanned excision and residual disease on re-excision specimens can adversely affect outcomes, underscoring the importance of meticulous preoperative planning and complete resection when feasible [12]. Endoscopic endonasal approaches, sometimes combined with open cranio-orbitozygomatic techniques, allow maximal safe resection while preserving neurovascular structures. A detailed understanding of cavernous sinus compartments, defined by the intracavernous carotid artery, improves interpretation of apparent invasion and guides surgical planning. Endoscopic surgery enables a compartment-based approach, and collaboration between ENT and neurosurgery facilitates safe resection of lesions with multidirectional extension [13,14]. The selection of the operative approach was guided by tumor size, extent, and direction of invasion. Endoscopic transsphenoidal surgery offers superior panoramic visualization, improved illumination, and angled views that facilitate access to parasellar and clival compartments, particularly in lesions with cavernous sinus involvement. Compared with microscopic techniques, endoscopic approaches have been associated with improved visualization of critical neurovascular structures and comparable or improved extent of resection, without increased morbidity. In cases with extensive lateral or posterior extension, a staged or combined approach may be required to achieve maximal safe debulking while minimizing surgical risk [15]. Our case demonstrates how integrating imaging, intraoperative findings, and surgical expertise allowed safe resection of a lesion with extensive parasellar involvement.

Histological analysis was decisive. PAs are adenohypophyseal tumors classified by hormone expression and transcription factors, showing monomorphic cells in sheets, nests, or cords. Rathke cleft cysts are lined by ciliated cuboidal or columnar epithelium with goblet cells. Chordomas, by contrast, arise from notochord remnants and display cords of tumor cells in a myxoid background with characteristic vacuolated “physaliphorous cells.” Immunohistochemically, they express S100, a calcium-binding protein seen in cells with neuroectodermal or chondroid differentiation; pan-cytokeratin, a broad cytokeratin marker indicating epithelial differentiation; epithelial membrane antigen, another epithelial-associated marker; and brachyury, a nuclear transcription factor that is highly specific for notochord-derived tumors and is considered the key diagnostic marker for chordoma [16]. In this case, immunohistochemistry confirmed PA (synaptophysin positivity, reticulin loss) and Rathke cleft cyst (AE1/AE3 epithelial lining). Tissue diagnosis completely changed the expected natural history, prognosis, surgical strategy, and long-term follow-up, underscoring that histopathology is indispensable when imaging is ambiguous.

This case highlights several important clinical points. First, clival lesions with extensive bone destruction should not automatically be presumed malignant, as PAs, although typically benign, can mimic chordomas radiologically. Second, radiologic tools such as the Knosp score are helpful but may overestimate cavernous sinus invasion. Finally, accurate diagnosis requires a multidisciplinary approach integrating radiology, intraoperative assessment, endoscopic evaluation, and histopathology. Only by combining these perspectives can clinicians avoid misclassification, overtreatment, and incorrect prognostication. Ultimately, this case reinforces that tissue confirmation is crucial for guiding management and tailoring follow-up strategies in complex skull base tumors.

This case report has several limitations. First, postoperative CT and MRI images were not available for direct visual comparison with preoperative imaging. Although detailed radiologic descriptions were documented, inclusion of postoperative imaging would have strengthened the visual demonstration of tumor resection and residual mass. Second, as a single case report, the findings cannot be generalized to all patients with clival or ectopic PAs. Finally, long-term follow-up is limited; although six-month postoperative imaging demonstrated stable findings, longer-term outcomes, including recurrence or functional recovery, remain to be determined. Despite these limitations, the case provides valuable insights into the diagnostic challenges, surgical management, and histopathological confirmation of rare ectopic pituitary lesions.

## Conclusions

This case highlights the diagnostic challenges posed by skull base lesions that exhibit aggressive radiologic features. Although imaging strongly suggested a clival chordoma, histopathologic evaluation ultimately confirmed a benign PA associated with a Rathke cleft cyst. The patient’s presentation with severe headaches in the absence of visual, neurological, or endocrine deficits illustrates that clinical findings may not reflect the apparent radiologic severity of clival lesions.

The key message of this report is that radiologic aggressiveness does not reliably predict malignant pathology. Ectopic or clival PAs may demonstrate extensive bone destruction, cavernous sinus involvement, and vascular encasement, closely mimicking malignant skull base tumors and creating a significant risk of preoperative misclassification. Definitive diagnosis in this case was achieved only through the integration of imaging, intraoperative findings, and histopathologic confirmation. This case adds to the limited literature by emphasizing radiologic-pathologic discordance in clival PAs and reinforces the importance of a multidisciplinary, tissue-based approach to guide appropriate management and prognosis.
